# The Importance of Hemostasis on Long-Term Cardiovascular Outcomes in STEMI Patients—A Prospective Pilot Study

**DOI:** 10.3390/jcm14155500

**Published:** 2025-08-05

**Authors:** Aleksandra Karczmarska-Wódzka, Patrycja Wszelaki, Krzysztof Pstrągowski, Joanna Sikora

**Affiliations:** 1 Research and Education Unit for Experimental Biotechnology, Department of Transplantology and General Surgery, Collegium Medicum, Nicolaus Copernicus University, 85-094 Bydgoszcz, Poland; akar@cm.umk.pl (A.K.-W.); wszelakipatrycja@cm.umk.pl (P.W.); 2 Department of Cardiology and Internal Medicine, Antoni Jurasz University Hospital No. 1 in Bydgoszcz, 85-094 Bydgoszcz, Poland; pstragowski.krzysztof@gmail.com

**Keywords:** STEMI, myocardial infarction, cardiovascular risk, hemostasis, platelets

## Abstract

**Background/Objectives:** Platelet activity contributes to myocardial infarction; inadequate inhibition is a risk factor for stent thrombosis and mortality. Inadequate platelet inhibition during treatment is an important risk factor for stent thrombosis and may be associated with increased mortality. This study assessed platelet and coagulation activity in post-MI patients, identifying parameters associated with adverse ST-elevation myocardial infarction (STEMI) outcomes over 3 years, to identify patients needing intensive secondary prevention. **Methods:** From 57 admitted patients, 19 STEMI patients were analyzed. Thromboelastography (TEG) and Total Thrombus Formation Analysis System (T-TAS) were used to assess hemostasis and coagulation. Selected laboratory parameters were measured for correlations. Major adverse cardiovascular events (MACEs) were defined as ischemic stroke, myocardial infarction, ischemic heart disease, thrombosis, and death from cardiovascular causes. **Results:** The group with MACEs was characterized by a faster time to initial clot formation and greater reflection of clot strength. T-TAS parameters, such as area under the curve at 10 min (T-TAS AUC10), showed lower values in the same group of patients. A moderate positive correlation suggested that as white blood cell count increases, T-TAS AUC10 values also tend to increase. A strong negative correlation (rho = −1.000, *p* < 0.01) was observed between low-density lipoprotein and kinetics in the TEG using the kaolin test at baseline in patients with MACEs. **Conclusions:** Some of the parameters suggest they are associated with adverse outcomes of STEMI, indicate the existence of an inflammatory state, and may contribute to risk stratification of STEMI patients and identify who will require ongoing monitoring.

## 1. Introduction

The routine classification of acute myocardial infarction (AMI) applied in everyday practice to facilitate the choice of treatment strategy is based on electrocardiographic findings and distinguishes ST-elevation myocardial infarction (STEMI) and non-ST-elevation myocardial infarction (NSTEMI) [1]. In STEMI, usually caused by acute total occlusion of a coronary artery, immediate primary percutaneous coronary intervention (PCI) is the mainstay of treatment [2]. The mainstay of treatment involves revascularisation of the culprit vessel/s to limit the area of irreversible necrosis. The myocardium continues to undergo a series of reparative processes to salvage the injured myocardium; numerous inflammatory cells are critical to the process of post-myocardial inflammation and remodelling [3]. Inflammation after a STEMI can significantly impact outcomes, including increased mortality and heart failure. Prolonged inflammation can lead to more cardiac inflammation, further damaging cardiomyocytes and increasing fibrosis, ultimately resulting in poor cardiac function [4]. Complementary to coronary revascularisation, dual antiplatelet therapy (DAPT), consisting of aspirin and P_2_Y12 receptor inhibitor [5]. Inadequate platelet inhibition during treatment with P_2_Y12 receptor inhibitors, defined as high platelet reactivity (HPR), is an important risk factor for stent thrombosis and may be associated with increased mortality [6].

Platelet activity is a major, but not the only, thrombosis-related mechanism that participates in myocardial infarction and influences subsequent prognosis. Platelets are responsible for the primary hemostasis, which consists of platelet adhesion, secretion, and aggregation [7]. Vascular injury and exposure of the von Willebrand factor initiate platelet adhesion and activation. As a result, the surface integrins—GP IIb/IIIa—gain high affinity to collagen and fibrinogen [8]. One of the most potent modulators of platelet function is adenosine diphosphate (ADP), the main agonist of platelet P_2_Y1 and P_2_Y12 receptors [9]. High platelet reactivity leading to clot formation inside the coronary arteries explains the pathomechanism of myocardial infarction and stent thrombosis—a possibly lethal complication of PCI with stent implantation [10].

Hemostasis is a dynamic, highly complex process involving many interacting factors, which include coagulation and fibrinolytic proteins, activators, inhibitors, and cellular elements [11]. The ideal way to treat a bleeding or prothrombotic patient is to measure the final product of the multitude of interacting factors and cellular components and their interactions in the shortest time possible. Hence, it is not easy to monitor and control its status over time. The key effect of antiplatelet therapy, which is implemented after coronary events, is to protect the patient from the future development of major adverse cardiovascular events (MACEs). This study investigates the impact of the description of primary hemostasis and coagulation cascade after primary PCI for STEMI on long-term follow-up beyond 3 years, which may help identify patients who could benefit from intensive secondary preventive care.

## 2. Methods

### 2.1. Study Design and Population

**Figure 1:**
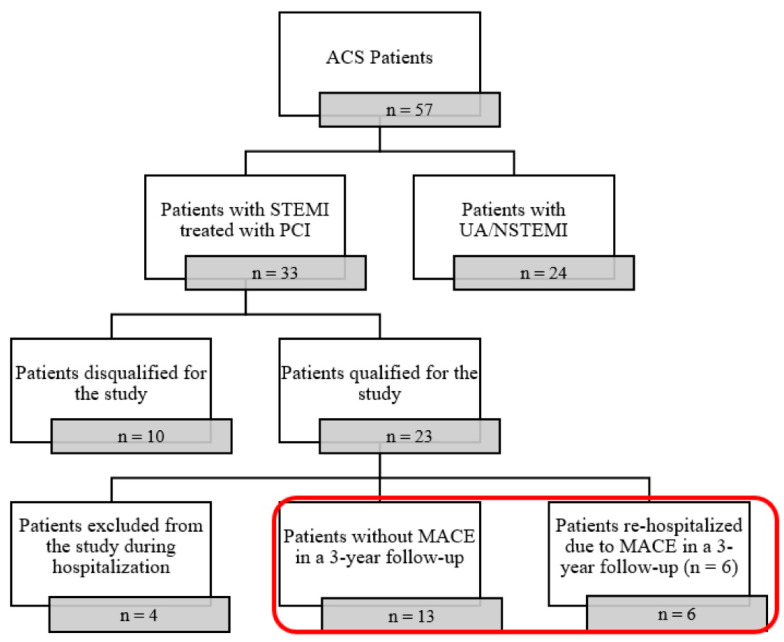
Trial flowchart. ACS: acute coronary syndrome; PCI: percutaneous coronary intervention; UA: unstable angina; STEMI: ST-segment elevation myocardial infarction; NSTEMI: non-ST-elevation myocardial infarction; MACE: major adverse cardiovascular events; n: number of patients.

The study was designed as a single-center, prospective, observational pilot study of consecutive patients with STEMI hospitalised between March and August 2017 in the Department of Cardiology and Internal Medicine in Jurasz Hospital No. 1 in Bydgoszcz. The analysis included only those patients who qualified for the study according to the inclusion criteria and participated in the study until the end of hospitalization. Detailed information about the inclusion/exclusion process of the study was presented in Figure 1.

The Declaration of Helsinki and Good Clinical Practice guidelines were followed in conducting the study. The protocol of the study was approved by the Ethics Committee of the Nicolaus Copernicus University in Toruń, Collegium Medicum in Bydgoszcz (approval number KB 89/2017; 17 January 2017). Each patient provided written informed consent to participate in the study before recruitment. All included patients immediately received 300 mg of aspirin and 180 mg of ticagrelor and were treated with PCI. Blood samples for assessment were drawn at three points: before drug administration, 1 day, and 5 days after inclusion into the study. The complete list of inclusion/exclusion criteria is presented in Table 1. Three years after enrolment in the study, we followed up patients to examine the long-term effects related to MACE, defined as any of the following events: stroke, myocardial infarction, heart failure, ischemic heart disease, thrombosis, or death from cardiovascular causes. The analysis included the first occurrence of any of these events.

### 2.2. Methods

According to the manufacturer’s instructions, platelet function testing was performed using thromboelastography (TEG5000) and Total Thrombus Formation Analysis System (T-TAS). Briefly, we used the T-TAS PL chip to measure platelet adhesion, release reaction, and platelet aggregation in totality, which we defined as a primary hemostatic response. Area under the pressure–time curve for 10 min, PL chip for T-TAS_AUC10 ≥260 indicates that primary hemostatic defects are not identified; however, T-TAS_AUC10 <260 is considered abnormal and indicates impaired primary hemostatic function (reduced platelet thrombus formation). Additionally, we checked the possibility of clot formation using TEG, using the kaolin test (CK) to initiate coagulation, where we verified each stage of clot formation (initiation, maximum amplitude, fibrinolysis). The pharmacodynamic evaluation was performed for each sample before drug administration and 1 day after. Biochemical variables from the blood were additionally analysed in a short follow-up on day 5 after myocardial infarction to assess the dynamics of changes using the standardized laboratory method in the Department of Laboratory Medicine in Jurasz Hospital, No. 1, Poland, in Bydgoszcz. We analyzed the following laboratory profiles: complete blood count parameters (white blood cell (WBC), red blood cell (RBC), hemoglobin test (HGB), hematocrit test (HCT), mean corpuscular volume (MCV), mean corpuscular hemoglobin (MCH), mean corpuscular hemoglobin concentration (MCHC), platelet count (PLT), red cell distribution width-standard deviation (RDW-SD), red cell distribution width-coefficient of variation (RDW-CV), platelet distribution width (PDW), mean platelet volume (MPV), platelet–large cell ratio (P-LCR), platelecrit (PCT), renal parameters (creatinine, modification of diet in renal disease (MDRD), blood urea nitrogen (BUN)), cardiac parameters (B-type natriuretic peptide (BNP), high-sensitivity troponin I (hsTnI)), coagulation parameters (activated partial thromboplastin time (APTT), prothrombin time (PT), prothrombin index (PT INR)), electrolytes (chloride, potassium, sodium), and inflammatory parameters (C-reactive protein (CRP)). The short time (5 days) over which we performed laboratory tests was planned due to the duration of the patients’ hospitalization.

### 2.3. Statistical Analysis

Pharmacodynamic, diagnostic, biochemical, and coagulation numerical variables are presented as mean ± standard deviation (SD), with additional descriptive information provided in brackets where appropriate. Categorical variables are presented as percentages. The Shapiro–Wilk test was used to assess the normality of distribution for numerical continuous variables. Depending on the distribution, group comparisons for numerical continuous variables were performed using the independent samples *t*-test or the Mann–Whitney U test. For categorical variables, group comparisons were conducted using the Chi-square test or Fisher’s exact test, as appropriate. Spearman’s rank correlation coefficients were calculated to explore relationships between variables where suitable. To assess changes in biochemical data across three time points within each group, the Friedman test was applied. Patient survival from the time of discharge was analyzed using Kaplan–Meier curves. For all analyses, a *p*-value of less than 0.05 was considered statistically significant. All statistical analyses were performed using SPSS Statistics, version 29.0.0.0.

## 3. Results

### 3.1. Study Population

Out of 57 patients who were admitted to the Department of Cardiology and Internal Medicine, 33 were diagnosed with STEMI and treated with PCI; 23 STEMI patients qualified for the study according to the inclusion criteria, four patients were excluded from the study during hospitalisation (one cardiogenic shock during coronary angiography, one qualified for coronary artery bypass graft (CABG), one necessity of abciximab administration, one death). Finally, we analyzed 19 patients, whom we divided into two groups after a 3-year follow-up, depending on the presence of MACEs. The details were as follows: one ischemic stroke, two ischemic heart diseases, one heart failure, one thrombosis, and one death. The study analyzed 19 patients, who were divided into two groups. Group 1 contained 13 patients without MACEs in a 3-year follow-up, whereas Group 2 contained 6 patients re-hospitalized due to MACEs in a 3-year follow-up.

The baseline characteristic is presented in the table below (Table 2). Shortly, the mean age and mean body mass index (BMI) were similar in both groups (mean age about 62–63 years, mean BMI about 27–28 kg/m^2^). The percentage of patients with hypertension was identical in both groups (61.5%). Both groups were characterized by a high rate of current smokers (about 80% in group 1 and 83.3% in group 2). It is worth noting the differences in the lipid profile: high-density lipoprotein (HDL) and triglycerides, which have unfavourable values in group 2 patients.

### 3.2. Pharmacodynamic

The pharmacodynamic assessment was performed in all study participants. Table 3 presents the mean ± SD for TEG parameters and T-TAS_AUC10 values at 0 and 24 h in patients with STEMI. The TEG parameters from the kaolin test include reaction time (R), kinetics (K), and maximum amplitude (MA). The results may indicate a decrease in R and K values from 0 h to 24 h in both groups, suggesting a faster clot formation and earlier achievement of clot strength following DAPT administration. TEG_CK_MA values increased from 0 h to 24 h in both groups, indicating enhanced clot strength. Coagulation parameters generally remained within the reference ranges, with no significant deviations from the observed norm. T-TAS_AUC10 values decreased at 24 h in both groups, demonstrating a significant reduction in platelet aggregation following DAPT administration. In both groups, the results regarding primary hemostasis are satisfactory, with significantly reduced platelet activity after DAPT.

**Figure 2:**
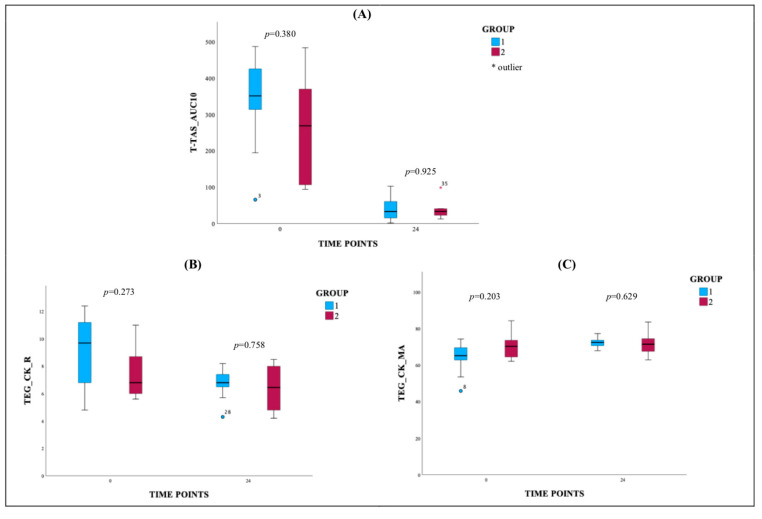
(**A**) T-TAS_AUC10, (**B**) TEG_CK_R, and (**C**) TEG_CK_MA. Graphical summary of T-TAS and TEG parameters; Group 1—patients without MACEs in a 3-year follow-up, Group 2—patients re-hospitalized due to MACEs in a 3-year follow-up. CK: kaolin test; R: reaction time; MA: maximum amplitude; T-TAS: Total Thrombus Formation Analysis System; AUC10: area under the pressure–time curve for 10 min, PL chip for T-TAS.

Figure 2 consists of three charts: (A) T-TAS_AUC10, (B) TEG_CK_R, and (C) TEG_CK_MA. This figure presents box plots illustrating the distribution of T-TAS_AUC10, TEG_CK_R, and TEG_CK_MA values in predefined time points. The T-TAS_AUC10 (chart A) shows a marked reduction in values at 24 h compared to 0 h in both groups. The TEG_CK_R plots (chart B) show a decrease in reaction time at 24 h, which indicates faster clot initiation. The TEG_CK_MA plots (chart C) demonstrate increased maximum amplitude at 24 h, indicating stronger clot formation.

### 3.3. Laboratory Analysis

Results of selected laboratory tests in the study population at three different time points during hospitalisation are presented in Table 4. Significant differences above the reference values were observed for the parameters hsTnI, CRP, BNP, and APTT. CRP is the highest on the second day, and this is visible for both groups. In both groups, we observed increased WBC ranges, the trend is decreasing. BNP indicates potential heart failure risk, and hsTnI at 0 h and 24 h confirms myocardial injury, with a decrease at follow-up. Coagulation parameters showed prolonged APTT at 0 h, particularly in Group 1, but normalized by 24 h. In comparison, PT and prothrombin time–international normalized ratio (PT-INR) were slightly elevated at 0 h and decreased over time. Electrolyte levels (chloride, potassium, sodium) were generally within the reference ranges. In conclusion, these biochemical data suggest an initial inflammatory response and myocardial injury in STEMI patients. However, this trend is visible for both groups.

### 3.4. Correlations

**Figure 3:**
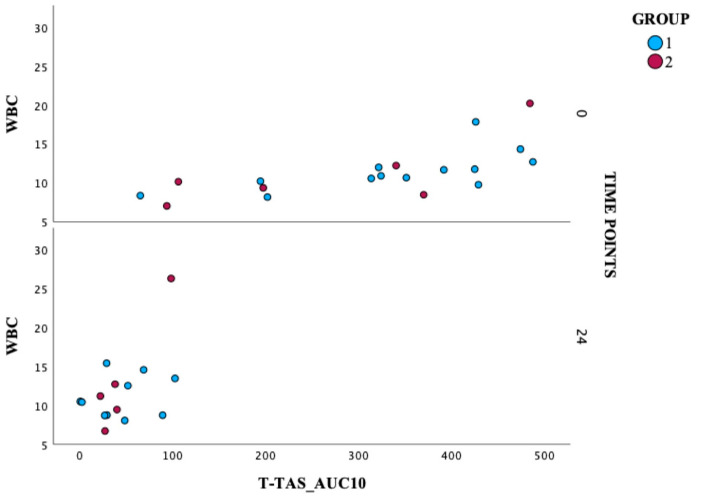
A scatter analysis of WBC and T-TAS_AUC10 levels. T-TAS: Total Thrombus Formation Analysis System; AUC10: area under the pressure-time curve for 10 min, PL chip for T-TAS; WBC: white blood cell.

This section presents the analysis of correlations between selected diagnostic parameters. These relationships may provide valuable insights into the pathophysiological mechanisms and potential biomarkers relevant to cardiovascular diseases. All correlations were evaluated using Spearman’s rank correlation coefficient (rho), a non-parametric method used to measure the strength and direction of the monotonic relationship between two variables. The value of rho ranges from −1 to +1, where values closer to either extreme indicate a stronger relationship. Figure 3 explores the relationship between WBC and T-TAS_AUC10 values. A moderate positive correlation was observed between baseline WBC and baseline T-TAS_AUC10 in Group 1 (rho = 0.687, *p* = 0.010). The spread of the points indicates a degree of variability in both WBC counts and platelet reactivity within the group. Despite the scatter, a general upward trend can be observed, which would require confirmation in a larger group of patients. This trend supports the moderate positive correlation, suggesting that as WBC count increases, T-TAS_AUC10 values tend to increase as well TAS_AUC10 values also tend to increase.

**Figure 4:**
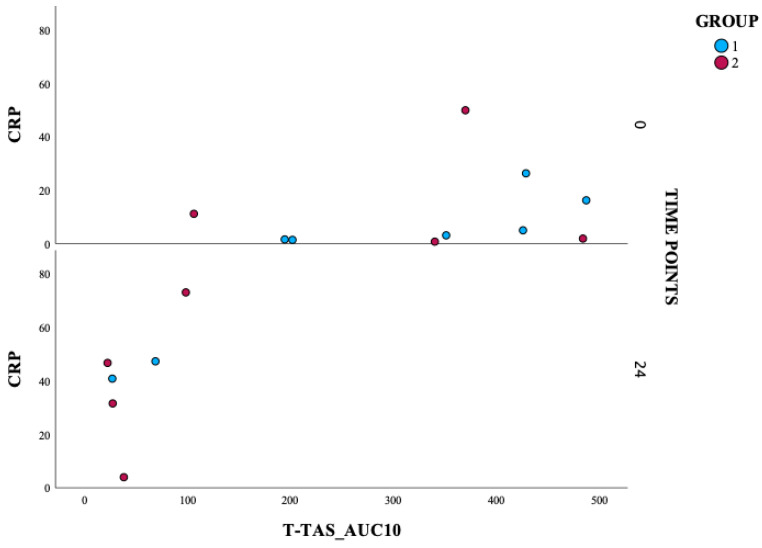
Correlation of CRP and T-TAS_AUC10 in two patient groups over time. T-TAS: Total Thrombus Formation Analysis System; AUC10: area under the pressure-time curve for 10 min, PL chip for T-TAS; CRP: C-reactive protein.

A scatter plot (Figure 4) was used to analyze the association between CRP and T-TAS_AUC10. A moderate positive correlation was observed between baseline CRP and baseline T-TAS_AUC10 in Group 1 (rho = 0.886, *p* = 0.019). This suggests that higher baseline CRP levels are associated with higher baseline T-TAS_AUC10 values in Group 1; this trend warrants further investigation.

**Figure 5:**
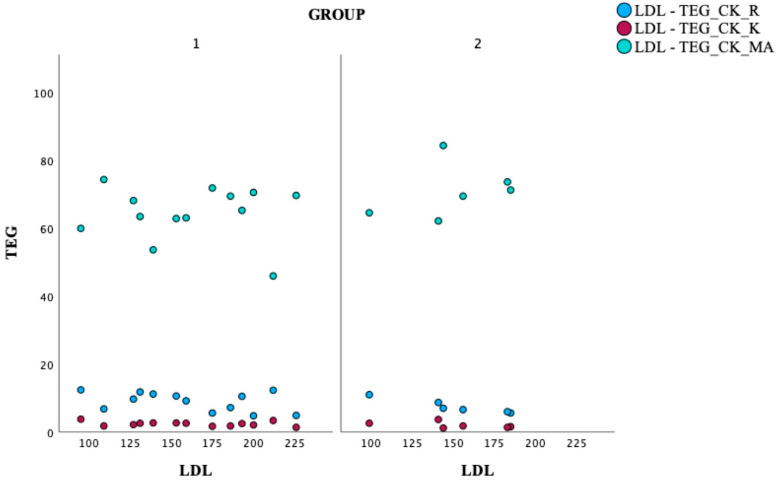
Visualizing the correlation between LDL and TEG parameters. TEG: thromboelastography; Group 1—patients without MACEs in a 3-year follow-up, Group 2—patients re-hospitalized due to MACEs in a 3-year follow-up. CK: kaolin test; R: reaction time, K: kinetics, MA: maximum amplitude.

The third part of the analysis (Figure 5) examines the association between LDL levels and clot formation parameters obtained through TEG. Group 2 found a strong negative correlation between LDL levels and the TEG_CK_K (rho = −1.000, *p* < 0.01). This suggests that higher LDL levels are associated with shorter clot formation time; this trend warrants further investigation. No significant correlations were observed between LDL levels and other TEG parameters, such as TEG_CK_R and TEG_CK_MA.

### 3.5. Kaplan–Meier Survival Analysis

A survival analysis was conducted on a cohort of 19 patients following STEMI to assess the time to rehospitalization over a 3-year observation period. During this period, 6 patients (31.6%) experienced rehospitalization (we defined it as MACE), while 13 patients (68.4%) were censored. MACE events occurred at various time points, with the cumulative proportion of patients remaining event-free being 0.947 at 8 days, 0.789 at 145 days, and 0.684 at 623 days. The Kaplan–Meier curve stabilized after the last event at 623 days, indicating a 68.4% probability of remaining free from MACEs beyond this point for patients still under observation. The estimated mean time to MACEs was 820.8 days (95% CI: 630.1–1011.6 days). The median time to MACEs was not reached, as the survival probability did not drop below 0.50 (the lowest observed survival probability for an event was 0.684). These findings suggest that a significant proportion of STEMI patients in this cohort did not require MACEs during the 3-year follow-up period (Table 5).

**Figure 6:**
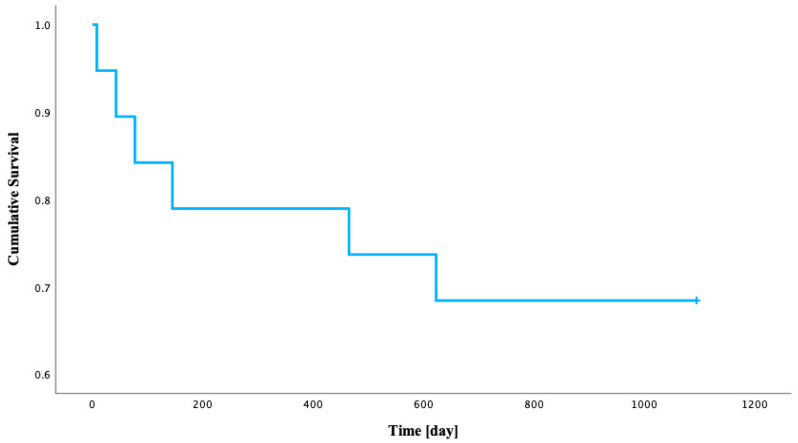
Kaplan–Meier estimated cumulative survival from rehospitalization over time in STEMI patients.

Figure 6 displays the Kaplan–Meier survival function, illustrating the estimated probability of patients remaining free from rehospitalization over time (in days from STEMI to MACE) following STEMI.

## 4. Discussion

Despite many effective therapies, the long-term risk of re-hospitalisation and mortality after STEMI remains high. In the era of routine primary PCI, studies evidenced a mortality rate of 7.3% at 1 year, and 2.05% per year thereafter [12]. The search for ways to identify patients at high risk of cardiovascular death who have little or no symptoms is still unclear. Patients who we could identify as being at higher risk for cardiovascular re-hospitalisation or death could benefit from advanced secondary prevention. Modern hypolipidemic treatment using PCSK9 inhibitors is effective for high-risk patients [13,14]. More intensive follow-up may focus better on managing blood pressure, diabetes mellitus, etc.

Most studies related to the prognosis analysis in STEMI are retrospective studies, based mainly on known factors concerning socio-demographic issues, age, gender, or risk factors for cardiovascular diseases such as obesity, smoking, etc. [15,16]. Previous studies have evidenced that the “pain-to-cathlab” time was a significant predictor for long-term cardiovascular mortality. Early emergency calls could shorten the time from pain to diagnosis in the event of chest pain. However, the quality of the time-period reported by the patients and physicians is questionable [17]. The latest reports showed [18] insights into intrahospital bleeding in STEMI. At the one-year follow-up, all-cause mortality, major bleeding, and reinfarction were significantly higher in the in-hospital bleeding group. Age, female sex, hypertension, and peripheral artery disease were found to be independent predictors of in-hospital bleeding, whereas drug-eluting stent implantation, radial access, and left ventricular ejection fraction were identified as protective factors. The incidents of major bleeding also indicate that it is worth remembering that not only is the inhibition of thrombosis important for these patients, but also a potential bleeding risk. Many studies showed that independent risk factors for all-cause mortality on follow-up beyond 6 months after pPCI for STEMI included advanced age, previous heart failure, renal dysfunction, and liver cirrhosis [19]; however in this study, we wanted to make sure that there would be no influence of comorbidities, so patients with previous cardiovascular events, kidney or liver disease were excluded from the study.

In the study, we focused on the coagulation cascade and primary hemostasis evaluation as a key reaction in clot formation during coronary artery occlusion, so we evaluated primary hemostasis using TTAS and coagulation cascade using TEG. We wanted to verify whether any parameters related to the mechanism of the hemostasis system, apart from platelet activity, may have long-term significance in assessing the occurrence of MACEs in a 3-year follow-up. The group in which MACEs occurred during the 3-year follow-up is characterized by a faster time to initial clot formation, greater reflection of clot strength, which may be related to the greater platelet activity of these patients, and a stronger effect of fibrin during clot formation. TTAS_AUC10, which shows the mechanism of primary hemostasis, is lower in this group of patients; this trend warrants further investigation in spite of the small group of patients.

The primary therapeutic goal in STEMI is early recanalization of the infarct artery to achieve myocardial reperfusion [20]. Rapid restoration of blood flow (during PCI) improves prognosis, reduces mortality, and preserves ventricular function [21]. Modulation of hemostasis, including antiplatelet and antithrombotic therapy, is a key component of pharmacological treatment for STEMI. However, other elements of the coagulation and platelet activation cascade are analyzed in the acute phase of myocardial infarction to discover the precise mechanisms of hemostasis that can become a therapeutic target. There is some evidence [22] that concentrations of t-PA antigen, D-dimer, and von Willebrand factor may be more modestly associated with first-ever coronary heart disease events. Factor XII also regulates the pathological process of thrombus formation on ruptured plaques. The degree of plaque disruption is associated with thrombus formation [23]. Specific arterial geometries resulting from atherosclerosis may enhance pathological thrombus formation after stenosis in a von Willebrand factor-dependent manner [23,24,25]. Research shows that cardiac magnetic resonance imaging can identify hemostasis-driven microvascular injury in STEMI, linking it to increased long-term mortality and major adverse cardiac events, with data revealing that platelet activation and thrombus formation are key factors in poor outcomes [26].

Additionally, we verified correlations with other processes associated with the occurrence of STEMI at that time, including lipid profile, CRP, and other selected laboratory parameters. The trend of the results supports the moderate positive correlation, suggesting that as WBC count increases, T-TAS_AUC10 values tend to increase as well. This finding may confirm a link between inflammation and platelet reactivity in these patients. Some reports have evidenced that the WBC count at admission provides effective predictors of poor outcome in patients with STEMI. In the study HORIZONS-AMI, STEMI patients undergoing primary PCI observed an independent association between leukocytosis, infarct size, and 1-year mortality [27]. The substudy of the INFUSE-AMI trial, in which the WBC at admission was an independent predictor of infarct size at 30 days on cardiac magnetic resonance imaging (MRI) in the same group of patients [28].

A strong negative correlation (rho = −1.000, *p* < 0.01) was observed between LDL and CK-K [min] at baseline in Group 2 patients. This may indicate that in patients experiencing re-hospitalization, elevated LDL levels are strongly linked to faster clot formation, as K time represents the time to clot formation in the TEG assay. The strength and consistency of this correlation suggest that LDL plays a significant role in influencing the coagulation profile in this patient population, potentially contributing to a prothrombotic state. Many studies analyse the correlation between lipid profile and mortality risk after STEMI [29]. However, we linked these parameters with clot formation. Gruber et al. [30] also tried stratifying the mortality risk in STEMI patients. In his retrospective study, elevated cholesterol and possibly low sodium levels were independent predictors of 1-year readmission after STEMI. These simple biomarkers can be considered for risk stratification of patients during outpatient follow-up of patients with STEMI.

Some clinical trials [31] such as CANTOS and COLCOT trials are extremely important in this context, as provide strong evidence that inflammation, a key factor in the development and destabilization of atherosclerotic plaque (leading to thrombotic events such as myocardial infarction and stroke), is an important therapeutic target, especially after an acute coronary event (including STEMI). Results from the CANTOS trial [32] showed a reduction in recurrent cardiovascular events in STEMI patients by targeting inflammation linked to hemostatic imbalances, showing a 15% reduction in major adverse events with canakinumab due to decreased IL-1β-mediated platelet activation. The COLCOT trial results [33] show that colchicine reduces cardiovascular events by 23% in STEMI patients through its anti-inflammatory actions, which modulate hemostasis by inhibiting neutrophil-platelet interactions, thereby lowering the risk of recurrent thrombosis and mortality over follow-up periods. This study underscores the interplay between inflammation and hemostasis, providing evidence for improved long-term outcomes with anti-inflammatory strategies in ACS. Although the literature does not provide direct evidence that CANTOS or COLCOT worked by directly affecting primary or secondary hemostasis, it clearly shows that modulating inflammation, which is the key process of atherothrombosis, effectively reduces thrombotic events in high-risk patients, including STEMI.

It is worth noting that the European Society of Cardiology (ESC) guidelines clearly recommend the use of more potent antiplatelet agents like ticagrelor and prasugrel in patients with ACS, limiting the use of clopidogrel to situations when newer agents are not available or contraindicated [34]. Some experts argue that the Federal Drug Administration was too fast to allow ticagrelor into the market with only one significant trial, PLATO, showing decreased mortality in treated patients [35]. The increased use of ticagrelor in antiplatelet management quantifies the need to establish whether this drug is better than the conventional DAPT. Still, some patients receive clopidogrel, which has a weaker antiplatelet effect; extending the study with an arm containing patients with a different DAPT strategy would be worthwhile.

### Study Limitations

Our study had some important limitations. First, a small count of the study group. This study was designed as a post hoc prospective research analysis, and as we do not influence the number of patients in long-term follow-up, power analysis was not performed. Next, we had patients with only one therapeutic strategy (DAPT-aspirin and ticagrelor). Finally, only one method of primary hemostasis and coagulation status assessment was used in the current analysis.

## 5. Conclusions

For the first time, we designed a prospective study that comprehensively characterizes STEMI patients in the acute phase in terms of platelet activity and the coagulation cascade, with patient survival with long-term follow-up. It is worth noting that we were able to indicate implications between hemostasis and inflammation. Even though platelet activity and the coagulation system are the most important pathophysiological processes observed after myocardial infarction, no significant predictive relationship could be confirmed in the short-term follow-up. However, some of the parameters suggest they are associated with adverse outcomes of STEMI, indicate the existence of a chronic inflammatory state, and may contribute to risk stratification of patients with STEMI and identification of those patients who will require ongoing monitoring. There are only preliminary results that require a study with a large group of patients. Further investigation is needed because we believe better compensation for inflammation or high lipid profiles may impact the overall outcome of patients. We suppose that modern treatment for patients with hyperlipidemia via PCSK9i [13,14] and sodium-glucose co-transporter two inhibitors (SGLT2i) [36] for patients with diabetes mellitus or heart failure may lead to a better cardiovascular outcome.

## Figures and Tables

**Table 1:** A complete list of inclusion and exclusion criteria for participation in the study.

**Inclusion criteria**
Men or non-pregnant women, aged 18–80 years
Provision of informed consent prior to any study-specific procedures
**Exclusion criteria**
Treatment with ticlopidine, clopidogrel, prasugrel, or ticagrelor within 14 days before the study enrolment
Current treatment with oral anticoagulant or chronic therapy with low-molecular-weight heparin
Active bleeding
History of intracranial hemorrhage
Recent gastrointestinal bleeding (within 30 days)
History of coagulation disorders
Platelet count less than <100 × 10^3^/mcL
Hemoglobin concentration less than 10.0 g/dL
History of moderate or severe hepatic impairment
History of major surgery or severe trauma (within 3 months)
Kidney disease requiring dialysis
Respiratory failure
History of severe chronic heart failure (NYHA class III or IV)
Concomitant therapy with strong CYP3A inhibitors (ketoconazole, itraconazole, voriconazole, telithromycin, clarithromycin, nefazadone, ritonavir, saquinavir, nelfinavir, indinavir, atazanavir) or strong CYP3A inducers (rifampicin, phenytoin, carbamazepine, dexamethasone, phenobarbital) within 14 days and during study treatment

NYHA: New York Heart Association Classification; CYP3A: Cytochrome P4503A.

**Table 2:** Baseline characteristics of trial participants.

Variable	STEMI Patients (n = 19)		
	Group 1 (n = 13) [%]	Group 2 (n = 6) [%]	*p*-Value
Male	61.5	83.3	0.605
Age in years	62.6 *	63.5 *	0.454
BMI in kg/m^2^	27.9 *	27.5 *	0.792
Hypertension	61.5	61.5	0.620
Active bleeding	0	0	-
History of transient ischemic attack	0	0	-
Chronic kidney disease	0	0	-
Hyperlipidaemia	45.5	0	0.487
Current smoker	80.0	83.3	1.000
History of smoking	23.1	0	0.517
Diabetes	33.3	0	0.509
Total cholesterol [mg/dL]	232.9 *	215.7 *	0.483
Cholesterol HDL [mg/dL]	55.4 *	47.2 *	0.114
Triglycerides [mg/dL]	103.1 *	113.2 *	0.693
Direct LDL cholesterol [mg/dL]	161.9 *	151.3 *	0.599
antigen-HCV	negative	negative	
antigen-HBs	negative	negative	
ALT [U/L]	28.4 *	23.0 *	0.655
AST [U/L]	39.0 *	24.0 *	0.653

Data are shown as a contribution percentage of patients from specific study group; *—data are shown as a mean; Group 1—patients without MACEs in a 3-year follow-up, Group 2—patients re-hospitalized due to MACEs in a 3-year follow-up.—BMI: body mass index; STEMI: ST-elevation myocardial infarction; HDL: high-density lipoprotein; LDL: low-density lipoprotein; ALT: alanine transaminase; AST: aspartate transferase; U: units; n: number of patients.

**Table 3:** TEG and T-TAS parameters at baseline and 24 h after DAPT administration.

Data	STEMI Patient [mean ± SD]					
	0 h			24 h		
	Group 1 (n = 13)	Group 2 (n = 6)	*p*-Value	Group 1 (n = 13)	Group 2 (n = 6)	*p*-Value
TEG_CK_R [min]	9.0000 ± 2.80	7.4833 ± 2.03	0.273	6.7846 ± 0.98	6.4000 ± 1.73	0.758
TEG_CK_K [min]	2.4077 ± 0.68	2.0500 ± 0.94	0.201	1.7385 ± 0.29	1.7667 ± 0.44	0.532
TEG_CK_MA [mm]	64.4231 ± 7.89	70.8500 ± 7.84	0.203	72.4769 ± 2.62	71.9167 ± 7.04	0.629
T-TAS_AUC10	339.0538 ± 123.03	265.6000 ± 157.25	0.380	41.1750 ± 33.16	40.1833 ± 30.53	0.925

Data are reported as mean  ±  standard deviation (SD); Group 1—patients without MACEs in a 3-year follow-up, Group 2—patients re-hospitalized due to MACEs in a 3-year follow-up. DAPT: dual antiplatelet therapy; STEMI: ST-elevation myocardial infarction; TEG: thromboelastography; CK: kaolin test; R: reaction time; K: kinetics; MA: maximum amplitude; T-TAS: Total Thrombus Formation Analysis System; AUC10: area under the pressure-time curve for 10 min, PL chip for T-TAS.

**Table 4:** Baseline biochemical variables of those enrolled in the study.

		STEMI Patient [mean ± SD]									*p*-Value ^a^	*p*-Value ^b^
		0 h			24 h			Follow Up				
Biochemical Data	Reference Range	Group 1 (n = 13)	Group 2 (n = 6)	*p*-Value	Group 1 (n = 13)	Group 2 (n = 6)	*p*-Value	Group 1 (n = 13)	Group 2 (n = 6)	*p*-Value		
WBC [10^3^/μL]	4.23–9.07	11.46 ± 2.54	11.24 ± 4.72	0.430	10.74 ± 2.83	13.27 ± 7.60	0.629	9.49 ± 1.58	7.88 ± 2.72	0.108	0.002	0.015
RBC [10^6^/μL]	4.63–6.08	4.71 ± 0.48	4.66 ± 0.51	0.861	4.35 ± 0.38	4.62 ± 0.37	0.532	4.07 ± 0.42	4.33 ± 0.39	0.421	<0.001	0.165
HGB [g/dL]	13.7–17.5	14.5 ± 1.38	14.5 ± 1.65	0.965	13.4 ± 1.01	14.1 ± 1.22	0.054	12.4 ± 1.17	13.4 ± 1.04	0.145	<0.001	0.091
HCT [%]	40.01–51.0	42.93 ± 4.03	42.92 ± 5.14	0.661	39.57 ± 2.95	42.10 ± 3.47	0.041	37.37 ± 3.21	39.67 ± 3.34	0.315	<0.001	0.022
MCV [fl]	79.0–92.2	91.3 ± 3.60	92.1 ± 3.37	0.599	91.1 ± 3.14	91.3 ± 4.48	0.910	91.9 ± 4.15	88.0 ± 2.52	0.161	0.236	0.223
MCH [pg]	25.7–32.2	30.8 ± 0.93	31.1 ± 1.51	0.508	30.7 ± 0.85	30.7 ± 1.69	0.910	30.6 ± 1.09	30.8 ± 1.62	0.745	0.035	0.143
MCHC [g/dL]	32.3–36.5	33.8 ± 0.72	33.8 ± 1.12	0.757	33.8 ± 0.65	33.6 ± 0.44	0.533	33.3 ± 0.81	33.7 ± 0.91	0.512	0.003	0.801
PLT [10^3^/μL]	132–370	251.0 ± 45.56	234.7 ± 40.17	0.273	231.9 ± 42.65	228.4 ± 37.54	0.650	236.5 ± 59.27	244.0 ± 39.02	0.763	0.050	0.819
RDW-SD [fl]	35.1–43.9	45.6 ± 3.89	47.2 ± 4.34	0.661	46.4 ± 3.66	47.3 ± 4.59	0.610	45.6 ± 3.89	46.6 ± 3.58	0.513	0.641	0.449
RDW-CV [%]	11.6–14.4	13.8 ± 0.73	14.2 ± 0.82	0.482	14.0 ± 0.73	14.2 ± 0.94	0.865	13.8 ± 0.71	13.9 ± 0.65	0.762	0.497	0.019
PDW [fl]	9.8–16.1	13.3 ± 1.94	13.6 ± 1.94	0.605	13.3 ± 2.50	13.1 ± 1.74	0.777	14.5 ± 3.25	12.9 ± 1.57	0.451	0.195	0.076
MPV [fl]	9.4–12.6	10.9 ± 0.89	11.1 ± 0.91	0.778	11.1 ± 1.05	11.0 ± 0.65	0.909	11.4 ± 1.17	10.9 ± 0.78	0.421	0.301	0.846
P-LCR [%]	19.2–47.0	32.6 ± 7.33	32.9 ± 6.64	0.888	33.3 ± 8.60	33.0 ± 5.62	0.955	35.2 ± 8.01	31.8 ± 7.05	0.482	0.417	0.449
PCT [%]	0.16–0.35	0.28 ± 0.04	0.26 ± 0.04	0.258	0.25 ± 0.04	0.25 ± 0.04	0.528	0.27 ± 0.06	0.27 ± 0.04	0.762	0.016	0.368
CRP	<5.00	8.9 ± 10.15	15.9 ± 23.13	1.000	44.1 ± 4.59	38.9 ± 28.90	0.643	29.5 ± 1.69	14.3 ± 6.31	0.083	---	0.264
MDRD [mL/min]	>60	81.4 ± 14.43	74.2 ± 20.64	0.380	97.5 ± 15.19	84.4 ± 15.81	0.156	93.5 ± 14.75	84.2 ± 19.82	0.366	0.015	0.021
BUN [mg/dL]	8.4–25.7	15.6 ± 4.85	19.1 ± 9.02	0.625	13.2 ± 3.39	21.5 ± 7.31	0.077	18.3	19.6	0.317	---	---
Creatinine [mg/dL]	0.70–1.30	0.87 ± 0.09	1.02 ± 0.20	0.103	0.75 ± 0.08	0.88 ± 0.09	0.014	0.84 ± 0.21	0.91 ± 0.18	0.317	0.067	0.057
BNP	100–500 high risk of HF	50.9 ± 35.49	160.8 ± 196.01	0.439	241.1 ± 111.54	246.9 ± 15.63	0.564	111.5 ± 6.51	-	-	---	---
hsTnI [ng/L]	<34.2	637.9 ± 1309.12	16,743.2 ± 25,761.01	0.861	24,954.8 ± 20,592.28	16,073.2 ± 18,167.80	0.327	8123.9 ± 9422.47	3414.2 ± 3546.96	0.448	0.018	0.174
APPT [s]	26.4–37.5	159.2 ± 170.33	100.8 ± 115.89	0.461	30.2 ± 3.19	30.0 ± 3.57	1.000	30.0 ± 4.53	28.6 ± 1.41	1.000	0.135	---
PT [s]	9.4–13.8	14.7 ± 1.58	13.5 ± 0.98	0.072	13.1 ± 1.03	12.1 ± 0.64	0.093	13.1 ± 0.95	12.4 ± 0.35	0.248	0.060	---
PT INR index	0.9–1.2	1.2 ± 0.14	1.1 ± 0.12	0.292	1.1 ± 0.09	1.0 ± 0.05	0.180	1.1 ± 0.12	1.0 ± 0.04	0.564	0.060	---
Prothrombin index [%]	70.0–130.0	83.8 ± 9.34	90.5 ± 8.47	0.292	91.3 ± 8.99	96.8 ± 4.38	0.180	92.3 ± 9.49	96.7 ± 3.53	0.564	0.060	---
Chloride [mmol/L]	98.0–110.0	105.5 ± 4.42	107.2 ± 3.90	0.568	105.2 ± 4.53	104.3 ± 2.25	0.668	107.6 ± 2.21	107.8 ± 2.20	0.762	0.093	0.050
Potassium [mmol/L]	3.5–5.0	4.0 ± 0.41	4.2 ± 0.75	0.692	4.1 ± 0.36	4.2 ± 0.23	0.392	4.5 ± 0.65	4.5 ± 0.22	0.223	0.014	0.268
Sodium [mmol/L]	136.0–145.0	141.6 ± 5.43	143.9 ± 5.67	0.334	137.9 ± 2.76	138.6 ± 1.76	0.734	140.4 ± 3.19	140.2 ± 0.99	0.651	0.097	0.041

^a)^ results from Friedman test for Group 1; ^b)^ results from Friedman test for Group 2; --- p cannot be determined; Group 1—patients without MACEs in a 3-year follow-up, Group 2—patients re-hospitalized due to MACEs in a 3-year follow-up. STEMI: ST-elevation myocardial infarction; WBC: white blood cell; HGB: hemoglobin test; HCT: hematocrit test; MCV: mean corpuscular volume; MCH: mean corpuscular hemoglobin; MCHC: mean corpuscular hemoglobin concentration; PLT: platelet count; RDW-SD: red cell distribution width-standard deviation; RDW-CV: red cell distribution width-coefficient of variation; PDW: platelet distribution width; MPV: mean platelet volume; P-LCR: platelet–large cell ratio; PCT: platelecrit; CRP: C-reactive protein; MDRD: modification of diet in renal disease; BUN: blood urea nitrogen; BNP: B-type natriuretic peptide; hsTnI: high-sensitivity troponin I; APPT: activated partial thromboplastin time; PT: prothrombin time; PT INR index: prothrombin time–international normalized ratio.

**Table 5:** Survival rates after STEMI in a 3-year follow-up/Kaplan–Meier Survival Estimates for Time to Rehospitalization Following STEMI.

Time [Day]	STEMI Group	
	Survival Rate [%]	Standard Error
8	94.7	0.051
43	89.5	0.070
77	84.2	0.084
145	78.9	0.094
465	73.7	0.101
623	68.4	0.107

## Data Availability

The raw data supporting the conclusions of this article will be made available by the authors on request.

## Abbreviations

ACS	acute coronary syndrome
ADP	adenosine diphosphate
ALT	alanine transaminase
AMI	acute myocardial infarction
APTT	activated partial thromboplastin time
AST	aspartate transferase
AUC10	area under the pressure–time curve for 10 min, PL chip for T-TAS^®^
BMI	body mass index
BNP	B-type natriuretic peptide
BUN	blood urea nitrogen
CABG	coronary artery bypass graft
CK	kaolin test
CRP	C-reactive protein
CYP3A	cytochrome P4503A
DAPT	dual antiplatelet therapy
ESC	European Society of Cardiology
HCT	hematocrit test
HDL	high-density lipoprotein
HGB	hemoglobin test
HPR	high platelet reactivity
hsTnI	high-sensitivity troponin I
K	kinetics
LDL	low-density lipoprotein
MA	maximum amplitude
MACE	major adverse cardiovascular events
MCH	mean corpuscular hemoglobin
MCHC	mean corpuscular hemoglobin concentration
MCV	mean corpuscular volume
MDRD	modification of diet in renal disease
MI	myocardial infarction
MPV	mean platelet volume
MRI	magnetic resonance imaging
NSTEMI	non-ST-elevation myocardial infarction
NYHA	New York Heart Association Classification
PCI	percutaneous coronary intervention
PCSK9	proprotein convertase subtilisin/kexin type 9
PCT	platelecrit
PDW	platelet distribution width
P-LCR	platelet–large cell ratio
PLT	platelet count
PT	prothrombin time
PT-INR	prothrombin time–international normalized ratio
R	reaction time
RBC	red blood cell
RDW-CV	red cell distribution width-coefficient of variation
RDW-SD	red cell distribution width-standard deviation
SD	standard deviation
SGLT2i	sodium-glucose co-transporter 2 inhibitors
STEMI	ST-segment elevation myocardial infarction
TEG	thromboelastography
T-TAS	Total Thrombus Formation Analysis System^®^
U	units
UA	unstable angina
WBC	white blood cell
vWF	von Willebrand factor
